# Capsule Retractor Extension Technique for Ectopia Lentis

**DOI:** 10.1155/crop/4416608

**Published:** 2026-10-05

**Authors:** Christopher Young-Jin Kim, Dooho Brian Kim

**Affiliations:** ^1^ McCallie School Chattanooga Tennessee USA; ^2^ Professional Eye Associates Dalton Georgia USA; ^3^ Medical College of Georgia Augusta Georgia USA gru.edu

## Abstract

**Introduction:**

The surgical correction of ectopia lentis can range from routine to extremely challenging and largely depends on the extent of zonulopathy. When the lens‐bag complex is more severe and inaccessible using standard techniques, the risk of complication increases.

**Case Presentation:**

This is a single patient, bilateral and sequential surgical case report in which we describe a technique to improve access to the lens‐bag complex when out of reach using standard surgical techniques. We describe a simple technique to extend the reach of a capsule retractor by stretching and extending the internal tip with microforceps to engage the capsulorhexis edge in the setting of ectopia lentis.

**Conclusion:**

The capsule retractor extension technique simplifies and bypasses the need for any additional instrumentation to improve centration of the lens‐bag complex for phacoemulsification of the subluxated crystalline lens. It is a versatile surgical maneuver, which can be used to facilitate placement of the capsule retractor within the capsular bag in the setting of severe zonulopathy. The capsule retractor extension technique is important because it promotes preservation of the lens‐bag complex and anterior hyaloid face, which reduces the risk of vitreoretinal complications.

## 1. Introduction

Ectopia lentis occurs when the crystalline lens is displaced due to asymmetric weakness or absence of zonular support. This can lead to vision loss from functional aphakia or induced visual aberrations from looking through the peripheral lens. Ectopia lentis can occur due to trauma, pseudoexfoliation, or from hereditary ocular or systemic conditions. Due to poor accessibility and instability, lens extraction and intraocular lens (IOL) placement are particularly challenging. The lack of zonular support makes it difficult to puncture the anterior capsule and perform a well‐sized and centered capsulorhexis. Moreover, the hyaloid face is more exposed, and the lens is partially hidden from view under the pupil, which increases the risk for anterior capsule tear, vitreous loss, dropped lens, and retinal detachment [1].

Ideally, the crystalline lens is removed with an IOL placed within an intact capsular bag and anterior hyaloid face. If necessary, a capsule tension ring (CTR) with or without a sutured capsule tension segment (CTS) is required to center the IOL‐bag complex [2]; however, in some cases, the severity of lens decentration is so extreme that the surgeon is unable to reach the capsulorhexis edge with standard instrumentation such as iris hooks or capsule retractors. As a result, the lens‐bag complex is sacrificed, the anterior hyaloid face ruptured after vitrectomy, and the IOL secured either with anterior chamber (ACIOL), iris‐suture fixation, iris‐claw fixation, scleral‐suture fixation, or scleral‐haptic fixation; however, this strategy in younger patients with a well‐formed vitreous carries a higher rate of retinal detachment [3], IOL dislocation [4–6], pupil capture [7], and late IOL decentration.

In‐the‐bag IOL placement for ectopia lentis has been shown to have a low complication rate [8] relative to IOL fixation in the absence of capsule support. Fan et al. [3] analyzed the outcomes of lensectomy and vitrectomy for ectopia lentis from Marfan′s and found over a 17% retinal detachment rate. Therefore, it is of utmost importance to preserve the anterior hyaloid face and capsular bag whenever possible.

We describe a new technique whereby the capsule retractor is extended and stretched to engage the capsulorhexis edge for cases of severe lens subluxation. This simple maneuver facilitates safe lens removal and preservation of the lens‐bag complex and anterior hyaloid face.

## 2. Case Presentation

A 22‐year‐old Caucasian male with long‐standing ectopia lentis in both eyes presented with worsening vision in both eyes. Past medical history is significant for mild refractive amblyopia. The patient is adopted and unaware of family history for ectopia lentis. Patient declined genetic testing.

Uncorrected distance visual acuity was 20/200−1 in the right eye and 20/80−1 in the left eye. Manifest refraction was −7.75+6.00x085 in the right eye with 20/60 and −6.75+6.00x080 in the left eye with 20/60. Intraocular pressure was 10 in the right eye and 11 in the left eye. Slitlamp examination revealed severe superonasal lens subluxation in both eyes, 50% superonasal subluxation with 8 clock hours of zonular loss in the right eye (Figure 1a) and 40% superonasal subluxation with 6 clock hours of zonular loss in the left eye (Figure 1b).

**Figure 1:**
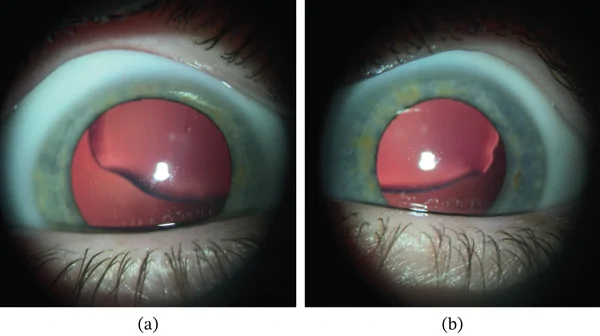
(a) Right eye with 50% superonasal lens subluxation and 8 clock hours of zonular loss. (b) Left eye with 40% superonasal subluxation and 6 clock hours of zonular loss.

Bilateral sequential lensectomy was achieved using the capsule retractor extension technique with CTR, scleral‐sutured CTS, and in‐the‐bag single piece acrylic IOL with a −1.50 target OU. Postoperative uncorrected distance visual acuity was 20/50 in the right eye and 20/60 in the left eye. Manifest refraction was −2.50+2.25x101 with 20/25 in the right eye and −3.75+3.25x037 with 20/30 in the left eye. Intraocular pressure was 8 in the right eye and 7 in the left eye.

There was no discernable endothelial cell loss after surgery. The right eye preoperative and postoperative endothelial cell count (Konan) was 3145 ± 76 and 3211 ± 79, respectively, whereas the left eye preoperative and postoperative endothelial cell count was 3247 ± 77 and 3215 ± 71, respectively (Figure 2). The patient has had 12 months of follow‐up with stable IOLs (Figure 3a,b) and no sign of complication.

**Figure 2:**
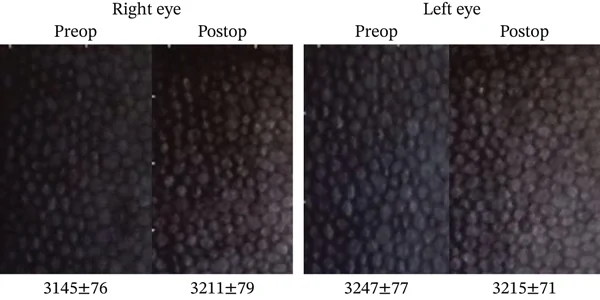
Right eye and left eye preoperative and postoperative endothelial cell counts.

**Figure 3:**
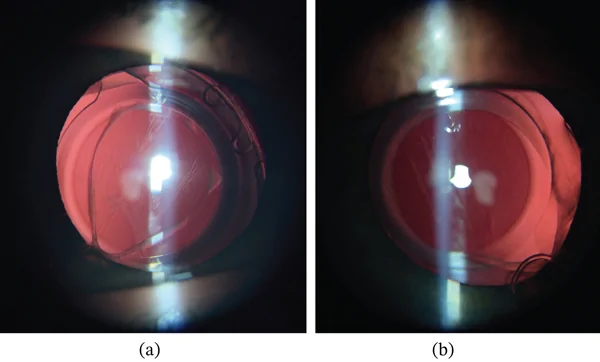
(a) Slitlamp examination of right eye 12 months after surgery demonstrating a well centered single‐piece acrylic IOL within the capsular bag. Observe the CTR eyelets visible at 2 o′clock and in good position. Note the posterior capsule opacification. (b) Slitlamp examination of the left eye 12 months after surgery demonstrating a well centered single‐piece acrylic IOL within the capsular bag. Observe the central CTS eyelet visible at 5 o′clock and in good position. Note the posterior capsule opacification.

## 3. Surgical Technique

Four to five clock hours of capsulorhexis are achieved and with the anterior chamber full of dispersive viscoelastic, a paracentesis incision is placed over the area of zonulopathy. A capsule retractor (#MCR‐0001, MicroSurgical Technology, United States) is placed through this incision. If the subluxated lens‐bag complex cannot be reached by the capsule retractor (Figure 4), microforceps are placed through a limbal incision 90° away from the capsule retractor and the tip is straightened and extended to hook around the capsulorhexis edge. Care is taken to avoid contact with the corneal endothelium by angling the capsule retractor sideways (Figure 5 and File S1). The external tip of the capsule retractor is secured with smooth forceps just proximal to the stopper to prevent the entire capsule retractor from slipping into the anterior chamber (Figure 5). Once the tip is tucked around the rhexis edge, the stopper is cinched down to pull the lens‐bag complex toward the center (Figure 6). The flexible nature of the capsule retractor allows it to be extended without damage. Once the lens‐bag complex is better centered, additional capsule retractors can be placed on either side of the first retractor through limbal stab incisions for more support (Figure 7), and capsulorhexis is completed (Figure 8).

**Figure 4:**
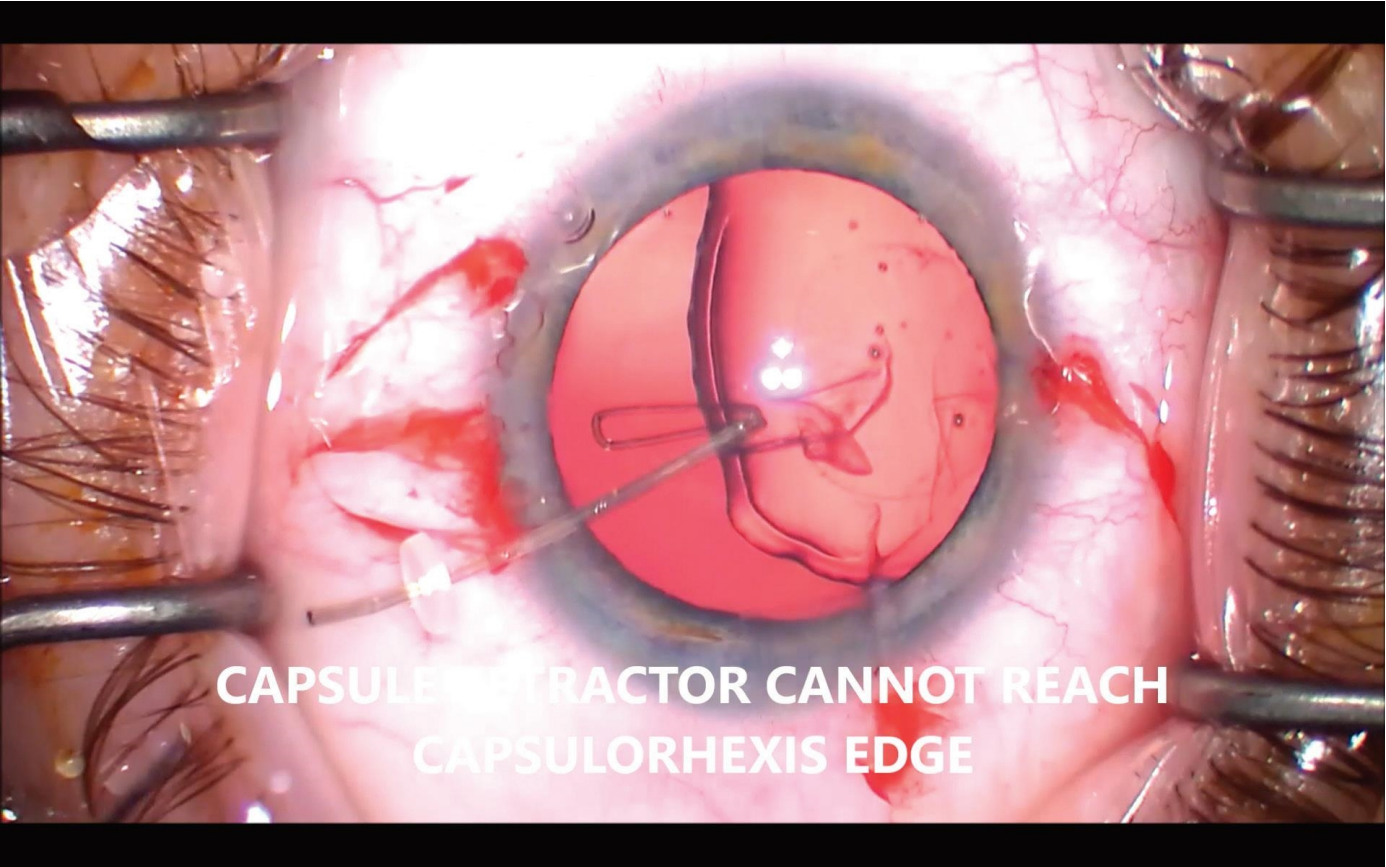
The capsule retractor is placed through a limbal incision. Observe that the capsule retractor even at its maximum length is unable to reach the capsulorhexis edge due to the severity in lens subluxation (from File S1, time stamp 15 s).

**Figure 5:**
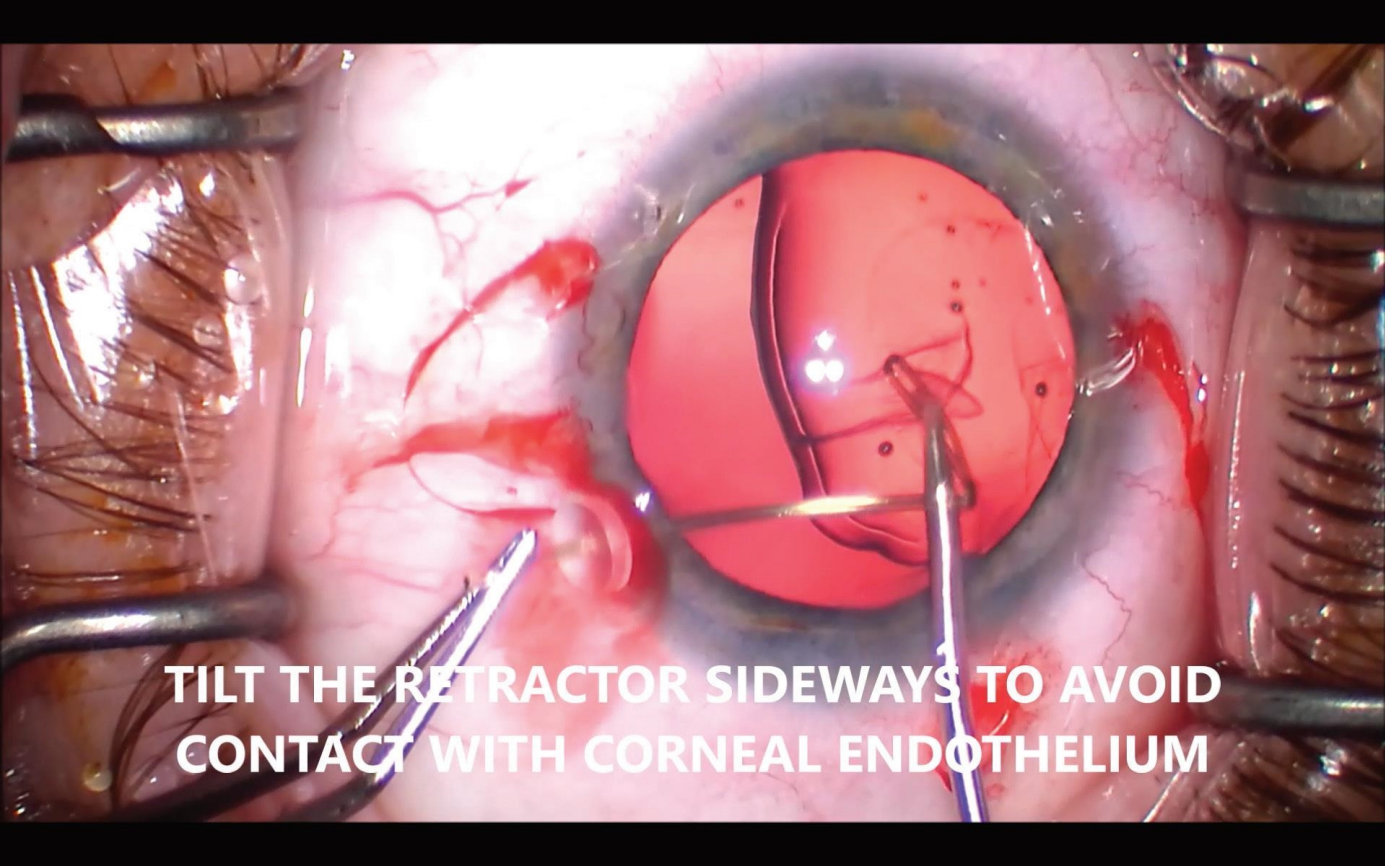
Using a two‐handed technique, the left‐hand forceps is used to secure the external tip of the retractor to prevent the entire retractor from slipping into the anterior chamber, whereas the right‐hand microforceps is used to straighten the elbow of the retractor effectively increasing its length. The capsule retractor tip is extended and slid under the capsulorhexis edge. Observe the retractor is turned sideways to prevent contact with the corneal endothelium (from File S1, time stamp 18 s).

**Figure 6:**
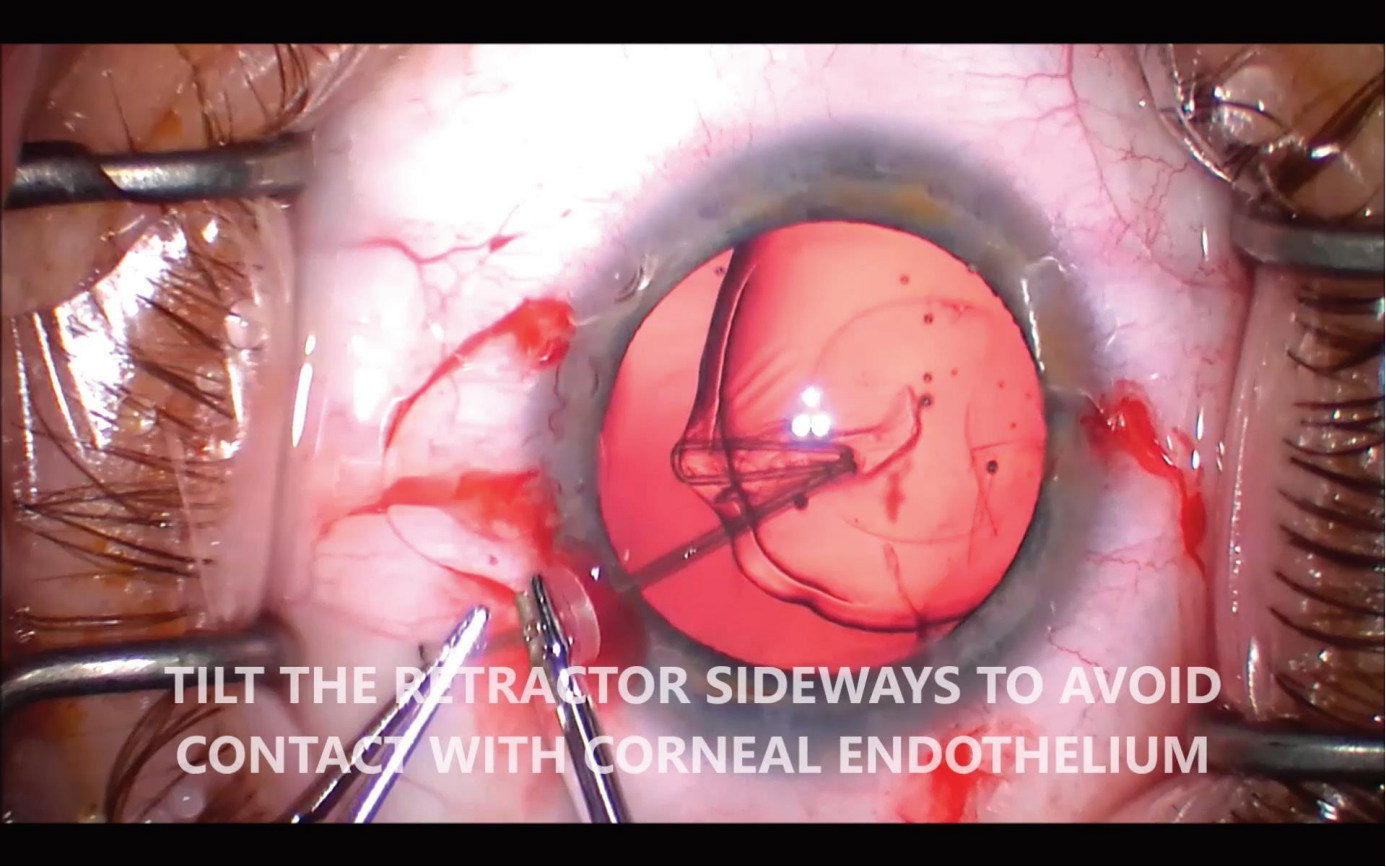
The capsule retractor stopper is cinched down, which improves centration of the lens‐bag complex. Observe the rounded tip of the retractor provides a broader base for gentle capsule fornix support (from File S1, time stamp 20 s).

**Figure 7:**
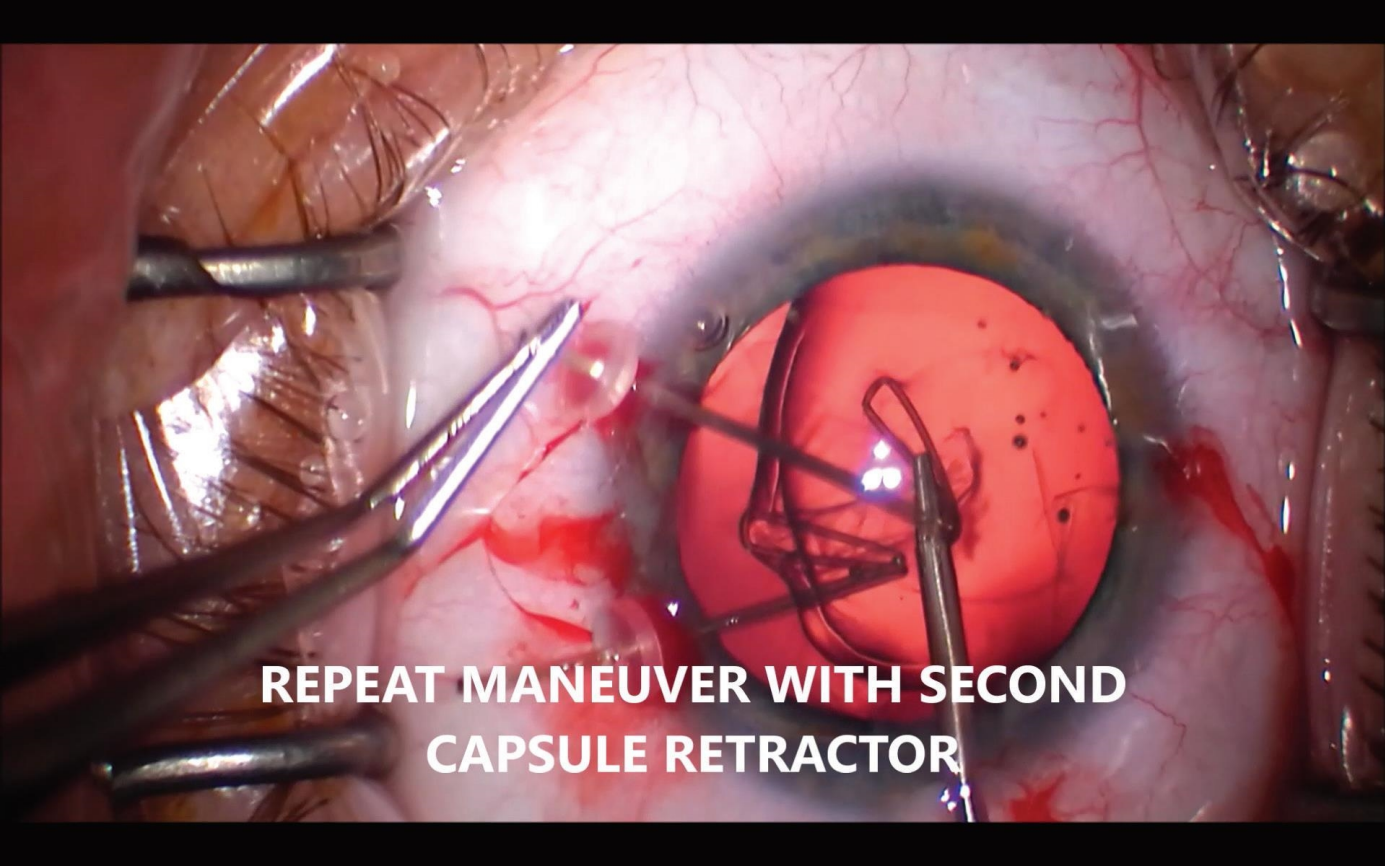
The same maneuver is performed on a second capsule retractor, extending the tip with microforceps around the capsulorhexis edge (from File S1, time stamp 22 s).

**Figure 8:**
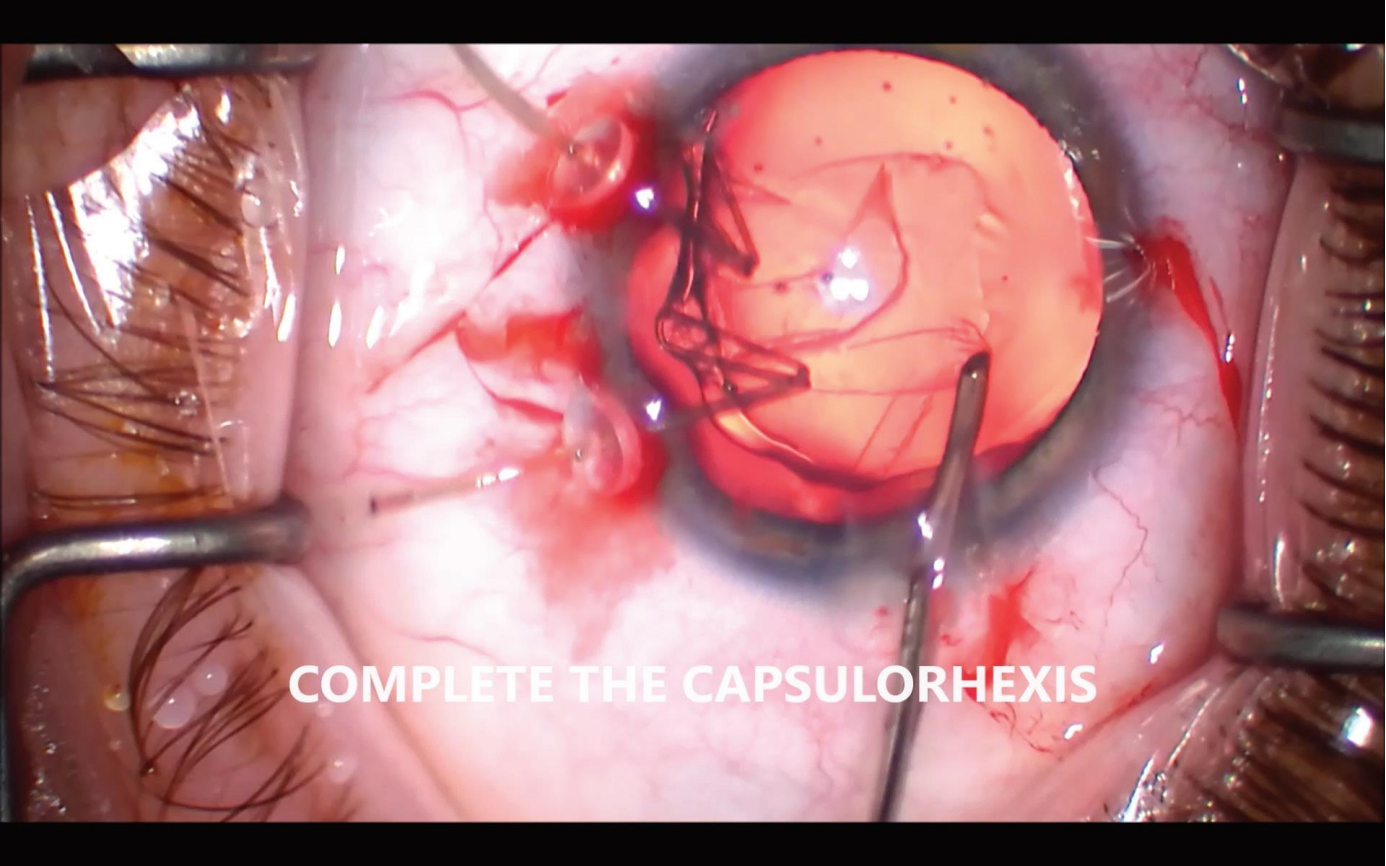
Both capsule retractors are cinched down to improve lens‐bag complex centration and capsulorhexis is completed (from File S1, time stamp 25 s).

A right‐angle hydrodissection cannula is used to separate the lens from the capsular bag. Mechanical irrigation and aspiration handpiece on low‐flow setting is used to aspirate the lens material. Cohesive viscoelastic is injected into the capsular bag followed by the CTR. The CTR diameter is compressed using a Lester hook through the leading eyelet. Capsule retractors are removed and a 6‐0 silk traction suture is placed through the limbus contralateral to the area of zonular dehiscence. A peritomy is created over the area of dehiscence and a 3‐mm partial thickness scleral groove is placed 1.5 mm posterior to and parallel to the limbus. Both ends of the scleral groove are pierced full‐thickness with a 1‐mm paracentesis blade. CV‐8 Gore‐Tex suture (Gore‐Tex, Texas, United States) is placed through the central eyelet of the CTS. Using microforceps, the CTS‐Gore‐Tex‐suture complex is placed through the main incision and tucked into the capsular bag under the area of zonular deficiency. Using two microforceps, one of the Gore‐Tex suture ends is grasped and placed into the main incision, whereas the other microforceps through one of the sclerotomies receives the suture end with a hand‐shake technique, and the suture end is externalized through the sclerotomy. The same maneuver is performed with the other Gore‐Tex suture end through the other sclerotomy and the suture is tied with a single throw to pull the lens‐bag complex toward the center. The single piece acrylic IOL is injected into the capsular bag. The Gore‐Tex suture tension is adjusted and tied in a 1‐1‐1‐1 configuration and the knot buried. The conjunctiva is reapproximated with 8‐0 vicryl suture, viscoelastic is removed, the wounds are hydrated, and the case is completed (File S2). The capsule retractor extension technique is performed on the right eye in similar fashion (File S3).

## 4. Discussion

Before the development of capsule retractors, iris hooks were used in cases of ectopia lentis to engage the capsulorhexis edge to help center the capsular bag; however, these hooks were problematic because they were not designed for this purpose [9]. Iris hooks do not provide any support for the capsule fornix and equatorial posterior capsule. In the setting of zonulopathy, this results in a floppy posterior capsule, which can be easily aspirated and torn by the phacoemulsification or irrigation and aspiration tip. Furthermore, iris hooks are thin and excessive traction on the capsulorhexis can cause the anterior capsule to tear [10]. Lastly, the iris hook tip is not rounded, which can potentially puncture the capsule.

In contrast, capsule retractors are specifically designed to stabilize the capsular bag by providing broad equatorial support of the capsule fornix for capsulorhexis and phacoemulsification. They are superior to iris hooks in multiple ways: designed with an angle so that the tip retracts along the same plane as the lens‐bag complex; the tip engages the capsule equator to provide circumferential support to the posterior capsule; the tip design is smooth and round with a broad base to engage the capsule fornix in an atraumatic fashion.

However, when lens subluxation is more severe, the capsule retractor may not be able to reach the capsulorhexis edge and further surgical manipulation with additional instruments would be required to pull the capsulorhexis edge closer to the capsule retractor tip to engage the capsulorhexis edge. This extra manipulation can be risky since pulling on the fragile anterior capsule can easily lead to an anterior capsule tear, which can run out posteriorly. Once the bag is compromised, pars plana lensectomy, vitrectomy, and an alternative IOL fixation strategy without capsule support would be necessary, but younger patients with well‐formed vitreous have an increased risk for vitreoretinal complications [3]. Therefore, it is critical that the lens‐bag complex is preserved at all costs to minimize long‐term complication.

We describe a new technique in which microforceps are used to effectively increase the length and reach of the capsule retractor to the capsulorhexis edge by stretching and extending the tip by force. This is only possible with plastic capsule retractors, which are designed to bend and flex (MicroSurgical Technology, United States). This technique does not apply to metal capsule retractors. We can take advantage of this characteristic by stretching the retractor without damaging it. It is important to note that the manufacturer does not promote the use of the capsule retractor to be stretched and extended in the way we describe, thus this should be considered an off‐label use of the device. Furthermore, these capsule retractors are for single‐use only and should not be reused since device deformation under repeated stretching could compromise structural integrity. Fortunately, in our experience, the stretching of the capsule retractor with this technique as a single‐use device has not resulted in complication, breakage, or failure of the capsule retractor.

The capsule retractor extension technique has limitations. If lens subluxation is so severe that the capsulorhexis edge is beyond the reach of the capsule retractor despite stretching and extending the tip, an alternative technique would need to be employed to pull the lens closer to the capsule retractor. The author (DBK) describes the chopper‐capsular fornix hook technique [11] to hook and pull the lens‐bag complex with a chopper toward the center for better access in extreme cases of lens subluxation. This effectively improves access to and preserves the lens‐bag complex for IOL implantation, but the maneuver could potentially damage zonules in the process.

The capsule retractor extension technique offers a simple solution to engage the capsulorhexis edge using a microforcep to extend the reach of the capsule retractor tip. Since this is a single patient, two‐eye case series, the results are insufficient to establish generalizability, safety, or reproducibility of the technique. A larger sample size would be needed for such analysis.

## Author Contributions

Both D.B.K. and C.Y‐J.K. equally contributed to design, acquisition of data, manuscript writing, and manuscript editing. C.Y‐J.K. contributed to creating figures and video editing. D.B.K. contributed to conception and critical revisions of the manuscript.

## Funding

No funding was received for this manuscript.

## Disclosure

All authors (D.B.K. and C.Y‐J.K.) have read and approved the final version of the manuscript, had full access to all of the data in this study, and take complete responsibility for the integrity of the data and the accuracy of the data analysis. Lead author D.B.K. affirms that this manuscript is an honest, accurate, and transparent account of the study being reported; that no important aspects of the study have been omitted; and that any discrepancies from the study as planned have been explained.

## Ethics Statement

Ethical approval is not required for this study in accordance with local or national guidelines.

## Consent

Written informed consent was obtained from the patient for publication of the details of their medical case and any accompanying images.

## Conflicts of Interest

The authors declare no conflicts of interest.

## Supporting Information

Additional supporting information can be found online in the Supporting Information section.

## Supporting information


**Supporting Information 1** File S1: (Left eye) The anterior capsule is punctured with a sharp cystitome needle. Partial capsulorhexis is performed with forceps over the visible portion of the lens. Partial hydrodissection is performed. Capsule retractor is placed through a limbal incision and is too short to reach the capsulorhexis edge. The left hand is used to hold the external tip of the retractor with forceps, while the right hand is used to simultaneously grasp and then straighten the internal tip of the retractor to extend its reach of the capsulorhexis edge. The internal tip is tilted sideways to prevent contact with the corneal endothelium and then slid under the capsulorhexis edge. Another capsule retractor is placed 1.5 clock hours away in similar fashion.

**Supporting Information 2** File S2: (Left eye) The lens is removed by mechanical irrigation and aspiration handpiece. CTR is inserted. A 4‐0 silk traction suture is placed contraincisional to the area of zonulopathy. Peritomy and sclerotomy are performed. CTS with CV‐8 Gore‐Tex suture is placed within the capsular bag and the sutured ends are externalized from the sclerotomies. The IOL is placed within the capsular bag. Gore‐Tex suture is tied, conjunctiva is closed with 8‐0 vicryl suture, and the case is completed.

**Supporting Information 3** File S3: (Right eye) The anterior capsule is punctured with sharp capsulorhexis forceps and a partial capsulorhexis is performed over the visible portion of the lens. Capsule retractor is placed through a limbal incision and is too short to reach the capsulorhexis edge. Microforceps are used to grasp the internal tip of the retractor and then straightened to extend the tip toward the capsulorhexis edge. The tip is tilted sideways to prevent contact with the corneal endothelium and then slid under the capsulorhexis edge. Another capsule retractor is placed 1.5 clock hours away in similar fashion.

## Data Availability

The data that support the findings of this study are available on request from the corresponding author. The data are not publicly available due to privacy or ethical restrictions.
